# Changes in soil bacterial community and functions by substituting chemical fertilizer with biogas slurry in an apple orchard

**DOI:** 10.3389/fpls.2022.1013184

**Published:** 2022-09-20

**Authors:** He Zhang, Yue Ma, Jianzhu Shao, Rui Di, Feng Zhu, Zhichang Yang, Jianshe Sun, Xueying Zhang, Chunyan Zheng

**Affiliations:** ^1^ College of Horticulture, Hebei Agricultural University, Baoding, China; ^2^ Key Laboratory of Agricultural Water Resources, Hebei Key Laboratory of Soil Ecology, Center for Agricultural Resources Research, Institute of Genetics and Developmental Biology, Chinese Academy of Sciences, Shijiazhuang, China; ^3^ University of Chinese Academy of Sciences, Beijing, China; ^4^ The Key Laboratory of Crop Genetics and Breeding of Hebei, Institute of Cereal and Oil Crops, Hebei Academy of Agricultural and Forestry Sciences, Shijiazhuang, China

**Keywords:** biogas slurry, soil microbial ecology, community composition, functional structure, apple

## Abstract

Growing concerns about the negative environmental effects of excessive chemical fertilizer input in fruit production have resulted in many attempts looking for adequate substitution. Biogas slurry as a representative organic fertilizer has the potential to replace chemical fertilizer for improvement of sustainability. However, it is still poorly known how biogas slurry applications may affect the composition of soil microbiome. Here, we investigated different substitution rates of chemical fertilizer with biogas slurry treatment (the control with no fertilizer and biogas slurry, CK; 100% chemical fertilizer, CF; biogas slurry replacing 50% of chemical fertilizer, CBS; and biogas slurry replacing 100% of chemical fertilizer, BS) in an apple orchard. Soil bacterial community and functional structure among treatments were determined using Illumina sequencing technology coupled with Functional Annotation of Prokaryotic Taxonomy (FAPROTAX) analysis. Leaf nutrient contents, apple fruit and soil parameters were used to assess plant and soil quality. Results showed that most of fruit parameters and soil properties were significantly varied in the four treatments. CBS treatment increased the contents of soil organic matter, alkali nitrogen and available potassium average by 49.8%, 40.7% and 27.9%, respectively. Treatments with biogas slurry application increased the single fruit weight, fresh weight, and dry weight of apple fruit average by 15.6%, 18.8% and 17.8, respectively. Soil bacterial community dominance and composition were significantly influenced by substituting of chemical fertilizer with biogas slurry. Biogas slurry application enhanced the relative abundance of some beneficial taxa (e.g. Acidobacteria *Gp5* and *Gp7*, *Parasegetibacter*) and functional groups related to carbon and nitrogen cycling such as chemoheterotrophy, cellulolysis, and nitrogen fixation. Soil available phosphorus and potassium, pH and electrical conductivity were identified having a high potential for regulating soil bacterial specific taxa and functional groups. This study showed that the proper ratio application (50%: 50%) of biogas slurry with chemical fertilizer could regulate soil bacterial composition and functional structure *via* changes in soil nutrients. The variations of bacterial community could potentially take significant ecological roles in maintaining apple plant growth, soil fertility and functionality.

## 1 Introduction

Apple (*Malus domestica* Borkh.) is an important fruit and cash crop worldwide, but the average yields differed greatly among the apple-growing countries (FAOSTAT, 2020). In order to obtain a high productivity, conventional agriculture relies on large amounts of chemical fertilizers, pesticides and herbicides inputs. The excessive chemical inputs have been generating environmental problems and reducing biodiversity of soil fauna and soil quality (Zhang et al., 2018; Dimkpa et al., 2020; Hou et al., 2021). Therefore, reducing the application of chemical fertilizer and exploring environment-friendly fertilizers are increasingly concerned.

Biogas slurry is a byproduct of anaerobic digestion of animal wastes and crop straws. It has been considered as an effective organic substance and partially replace chemical fertilizer in agricultural and horticultural productions (Walsh et al., 2012; Wentzel et al., 2015; Ren et al., 2020; Tang et al., 2021). Biogas slurry contains high levels of nutrients of nitrogen(N), phosphorus (P), potassium (K) and other trace elements (Zhang et al., 2018; Ren et al., 2020). Furthermore, biogas slurry is also rich in amino acids, growth hormones, and effective microorganisms which could promote plant growth and stress tolerance (Bachmann et al., 2011; Insam et al., 2015; Ren et al., 2020; Tang et al., 2021). Previous studies showed that the application of organic materials in combination with chemical fertilizer could improve soil nutrient status, increase plant productivity and quality, decrease greenhouse gas emission while compared with application of raw chemical or organic materials (Zhang et al., 2018; Celestina et al., 2019; Bai et al., 2022). However, it also observed that there had no beneficial effect generating by the combination of chemical and organic fertilizer due to the different vegetation and soil types (Young et al., 2021). In addition, excessive application of biogas slurry might cause the pollution of groundwater and surface water and the accumulation of heavy metal and organic pollutant (Insam et al., 2015; Wang et al., 2019; Seleiman et al., 2020; Sobhi et al., 2022). Therefore, it is necessary to further study the proper ways of chemical fertilizer substitution using biogas slurry in agroforestry systems.

Soil microbiota play vital roles in ecosystem functions and processes, such as organic matter decomposition, soil nutrients cycling and regulating plant immune responses, thus soil microbiomes become widely used indicators for soil quality assessment (de Menezes et al., 2017; Thakur and Geisen, 2019; Delgado-Baquerizo et al., 2020). Previous studies have reported that biogas slurry application significantly affected the structure of soil bacterial community in comparison to using chemical fertilizer. Biogas slurry seems to influence soil bacterial community in a functional group specific manner, e.g. biogas slurry application has increased the relative abundance of methanogens and acetogenic bacteria while had no significant effect on eubacterial community (Insam et al., 2015). Additionally, applying biogas slurry in combination with chemical fertilizers at appropriate rate could influence some bacterial functional groups (e.g. ammonia-oxidizing bacteria, plant growth promoting bacteria), but the whole bacterial population was observed to have positive correlations with *Fusarium* wilt disease incidence (Cao et al., 2016). Yet, it is still unclear how biogas slurry substitution systems affect the structure and function of soil bacterial community. In consideration of productivity and quality of apple, there is a need to investigate the proper substitution ratio and overall effects of biogas slurry application in field condition.

In the present study, a field experiment was carried out to investigate the response of soil chemical properties, plant physiological characteristics and soil microbiome to the increment of replacing rate by biogas slurry. We hypothesized that: 1) characteristics of plant and soil parameters, and soil bacterial community and function are varied due to the different application ratio of chemical fertilizer with biogas slurry; 2) changes in soil bacterial community composition and function are related to the soil and plant parameters which were altered by the substitution of chemical fertilizer with biogas slurry.

## 2 Materials and methods

### 2.1 Study site

The study site was located at the Demonstration Station for Fruit Cultivation of Hebei Agricultural University, in Baoding City (Hebei Province, China, 38°97′99″N, 114°92′10″E, and 242 m a.s.l.). This area belonged to the foothills of the Taihang Mountain, and characterized by a warm temperate continental monsoon climate, with a mean annual temperature of 13.2°C and precipitation of 516 mm in 2019. The top soil (0-20 cm) was characterized for soil organic matter of 12.7 g kg^-1^, total nitrogen (N) of 0.77 g kg^-1^, total phosphorus (P) of 0.61 g kg^-1^, and total potassium (K) of 18.9 g kg^-1^ at the beginning of the experiment.

### 2.2 Experimental design and sampling

The study was carried out on 16th March 2019 in an apple orchard which was dominated by 5-year old Fuji trees (*Malus pumila* Mill.). The plant density was 1350 plants ha^-1^ with 4 m line spacing and 1.5 m plant spacing. The biogas slurry was collected from the demonstration station, where anaerobic digestion of cow manure was accomplished using a thermostatic anaerobic reactor. The digestate was subsequently separated into biogas slurry and biogas residue by two stages of slag removal and drying in a press separator. The properties of filtered biogas slurry were: pH 7.65, organic matter 5.02 g L^-1^, total N 1.42 g L^-1^, ammonia N 203.7 mg L^-1^, total P 0.46 g L^-1^, total K 0.93 g L^-1^, the contents of Fe, Mn, Zn, B, Mo were 29.06 mg L^-1^, 1.45 mg L^-1^, 0.81 mg L^-1^, 2.87 mg L^-1^, and 0.33 mg L^-1^, respectively.

The experiment consisted of four treatments: 1) the control with no fertilizer and biogas slurry (CK); 2) 100% chemical N fertilizer (CF); 3) biogas slurry replacing 50% of chemical N fertilizer (CBS); and 4) biogas slurry replacing 100% of chemical N fertilizer (BS). Each treatment was repeated three times in separated field plots. The four fertilization schemes were applied with the same amount of pure N. The insufficient supply of P and K nutrients in CBS and BS plots were compensated by proportional chemical fertilizer (including urea, diammonium phosphate and potassium dihydrogen phosphate; **Table 1**). Fertilizer application of the experiment was adopted the integrated management of water-fertilizer which chemical fertilizer and biogas slurry were mixed in irrigation water and equivalently applied in different stages of apple growth (before germination, flowering to fruitlets set, young fruit expansion and postharvest). To ensure the affordability of the young apple tree and comparability among the different treatments, we retained the same number of apple fruit during the growing season. The control of weeds and pests was followed the local general management.

**Table 1:** Amounts of chemical fertilizers, biogas slurry and irrigation applied in the experiment.

Treatment	Biogas slurry (m^3^ ha^-1^)	Chemical fertilizer (kg ha^-1^)			Irrigation (m^3^ ha^-1^)
		Nitrogen (N)	Phosphorus (P as P_2_O_5_)	Potassium (K as K_2_O)	
CK^1^	0	0	0	0	900
CF	0	150	75.0	150	900
CBS	52.8	75.0	50.7	101	847
BS	105.9	0	26.4	49.1	794

^1^CK, control, no chemical fertilizer and biogas slurry; CF, 100% chemical nitrogen (N) fertilizer; CBS and BS, biogas slurry replacing 50% and 100% of chemical N fertilizer, respectively.

Plant and soil samples were collected on 30th October, 2019, before the apple harvest. For each treatment, three replicates of three abreast trees were selected for the sampling of leaves, fruits, and soil in a plot. Firstly, we choose three abreast trees in each plot, and collected 30 healthy leaves with no sign of disease or nutrient deficiencies and 30 intact apples from the four directions of each tree, respectively (resulting in 90 leaves and 90 apples per plot). Collected leaves were fixed at 105°C and dried to a constant weight at 65°C before chemical analysis. Soil was collected at 0-20 cm depth under the canopy approximately 0.5 m from the trunk of the three abreast trees and pooled into one composite sample. Soil samples were put in an ice box and immediately carried to the laboratory. Each soil sample was divided into two subsamples: one part was kept at -80°C for determining soil bacterial community and the other part was sieved and air dried for the determination of soil parameters.

### 2.3 Plant and soil parameters analysis

#### 2.3.1 Leaf nutrients and fruit parameters

To effectively reflect the response of apple growth to the biogas slurry replacing of chemical fertilizer, the leaf nutrient contents (N, P and K) and fruit parameters (fruit weight and yield, solids content, soluble solids and malic acid) were analyzed. The contents of total N, P and K of leaf were measured using semimicro Kjeldahl method, Molybdenum blue colorimetry, and Flame spectrophotometer method, respectively. Fresh fruit weight and the dried pulp were determined by using a digital scale sensitive to 0.01 g, and the solids content was calculated. Soluble solid content was measured in juice by using a sugar analyzer (model PAL-1, ATAGO CO., LTD, Tokyo, Japan) and malic acid content analysis was done by using an acidometer (model GMK-835F, G-Won Company, Korea).

#### 2.3.2 Soil parameters

Soil organic matter and available N were measured using the potassium dichromate method and alkali nitrogen proliferation method, respectively. Soil pH and electrical conductivity (EC) were determined with soil to water ratio of 1:2.5 and 1:5, respectively. Soil available P concentration was determined by using modified molybdenum antimony anti-colorimetric method while soil available K was measured by flame photometer. The above parameters were analyzed following the procedures described by Bao (Bao, 2008) and soil porosity was evaluated followed the methods described by Wang et al. (Delgado-Baquerizo et al., 2020).

### 2.4 DNA extraction, PCR amplification and high-throughput sequencing

The total DNA was extracted from 0.5 g of fresh soil using the DNeasy PowerSoil Isolation Kit (Qiagen, Hilden, Germany) following the manufacturer’s protocol. The quality of DNA extracts was determined by the NanoDrop One spectrophotometer (Thermo Fisher Scientific, Waltham, MI, USA). We used the primer set 515F (5´- GTGCCAGCMGCCGCGGTAA-3´) and 806R (5´- GGACTACHVGGGTWTCTAAT-3´) to amplify the bacterial 16S rRNA gene from all soil samples (Caporaso et al., 2011). To avoid the possibility of false-positive PCR results, we used the ultrapure water as a negative control throughout the extraction and amplification processes. After amplification, the PCR products were purified, pooled and sequenced on an Illumina platform at the Novegene Bioinformatics Technology Co., Ltd. (Beijing, China).

### 2.5 Sequencing data processing

The bacterial raw sequences were merged using FLASH version 1.2.7 (Magoč and Salzberg, 2011) and assigned to each sample according to their barcodes. The resulting sequences were assigned to the same operational taxonomic unit (OTU) at the similarity of 97%. For each representative sequence, the Silva Database (http://www.arb-silva.de/) was used to annotate taxonomic information. OTUs abundance information was normalized using a standard of sequence number corresponding to the sample with the least sequences, and diversity indices (Chao1, Shannon diversity and Berker-parker index) were calculated using the vegan package. We used the Functional Annotation of Prokaryotic Taxonomy (FAPROTAX) database to analyze the functional groups of bacteria in the soil with default settings in the output functional table (Louca et al., 2016). The raw data have been stored in the NCBI database under accession number PRJNA875317.

### 2.6 Statistical analysis

The Shapiro-Wilk and Bartlett’s tests were used for the normality test and checking the homogeneity of variances, respectively. We used linear mixed effect model (LMM) with Tukey’s multiple comparison to evaluate the effects of the biogas slurry as substitution for chemical N fertilizer on soil and plant parameters (soil physico-chemical parameters, leaf nutrients and apple fruit parameters), soil bacterial diversity (Chao1, Shannon diversity and Berker-parker index) and functional groups. The different fertilizer rate was used as a fixed effect and the specific number of plot as the random effect. The analysis was performed using the *lme4* (Bates et al., 2015), *lmerTest* (Kuznetsova et al., 2017) and *multcomp* (Hothorn et al., 2008) packages in R (version 4.1.2). We used principal coordinate analysis (PCoA) based on Bray-Curtis dissimilarity to detect the variation in bacterial community composition and functional structure (carbon, nitrogen and sulfur cycle, respectively), followed by a permutational multivariate analysis of variance (PERMANOVA) with 999 permutations using the *vegan* package. The Statistical Analysis of Metagenomic Profiles (STAMP, version 2.1.3) was used to determine the significantly different bacterial taxa and functions among the substitution sequence of biogas slurry with chemical N fertilizer (CF VS CK, effect of chemical N fertilizer; CBS VS CF, effect of biogas slurry replacing 50% of chemical N fertilizer; and BS VS CBS, effect of biogas slurry replacing 100% of chemical N fertilizer). We used redundancy analysis (RDA) to elucidate the relationships between the bacterial community composition or functional structures and soil or plant parameters, respectively, and the relations were examined using the Monte Carlo permutation (9999 repetitions). Further, Spearman correlation coefficients were calculated to reveal the relationships among soil physico-chemical parameters, leaf nutrients, and apple fruit parameters with the bacterial diversity indices, the relative abundances of specific abundant taxa and functional groups. Network was used to visualize the relationships of soil and plant parameters. The data used in network construction conformed to irhoi > 0.7 and p < 0.05 in the Spearman correlation analysis, and the network plots were generated by using Gephi (0.9.2).

## 3 Results

### 3.1 Leaf nutrients, fruit parameters and soil properties

The results indicated that most of fruit parameters and soil properties were significantly varied in the four treatments (**Table 2**). The contents of soil organic matter, alkali N and available K, and soil porosity were higher in the CBS treatment than in the CK, CF and BS treatments (p < 0.001). The soil available P was significantly increased under fertilized treatments by 66.9-152.3% compared to the CK treatment. We found that CBS treatment has the lowest pH with a minimum value of 6.54, and CF treatment has the lowest EC with a value of 54.77. CBS and BS treatments significantly increased the single fruit weight, fresh weight, and dry weight of apple fruit compared to CK and CF treatments (p < 0.05). However, the proportion of soluble solids in CBS and CF treatments was lower than that in CK treatment but showed no difference from the BS treatment. No significant differences were observed in the leaf nutrients, dry matter content, malic acid percent and the solid acid ratio of apple fruit among the four treatments (p > 0.05).

**Table 2:** Soil physico-chemical parameters, leaf nutrients and fruit parameters of apple in different treatments.

Index	Treatment				Linear mixed effects model		
	CK^1^	CF	CBS	BS	*R^2^*	*F*	p
Soil physico-chemical parameters							
Soil organic matter (g kg^-1^)	10.27 ± 0.43c^2^	12.99 ± 0.74b	18.40 ± 2.02a	10.32 ± 0.92c	0.92	35.89	**< 0.001**
Alkali N (mg kg^-1^)	62.05 ± 5.17b	72.76 ± 8.76b	101.9 ± 9.42a	64.32 ± 2.65b	0.91	35.16	**< 0.001**
Available P (mg kg^-1^)	18.41 ± 1.20c	46.45 ± 8.36a	39.59 ± 10.23ab	30.73 ± 1.78b	0.83	18.47	**0.002**
Available K (mg kg^-1^)	124.80 ± 0.58c	129.16 ± 1.83c	182.16 ± 7.42a	146.01 ± 3.45b	0.98	115.42	**< 0.001**
Soil porosity (%)	54.83 ± 1.10b	53.81 ± 0.56b	58.41 ± 0.97a	55.18 ± 0.47b	0.87	17.55	**< 0.001**
pH	7.84 ± 0.08a	6.99 ± 0.29b	6.54 ± 0.03c	7.76 ± 0.04a	0.95	51.72	**< 0.001**
Electrical conductivity (EC)	79.03 ± 12.77a	54.77 ± 2.80b	68.90 ± 7.52ab	79.57 ± 5.04a	0.71	6.41	**0.016**
Leaf nutrients							
Leaf total N (g kg^-1^)	18.77 ± 1.70a	20.08 ± 1.36a	17.13 ± 2.31a	16.74 ± 2.07a	0.43	1.99	0.194
Leaf total P (g kg^-1^)	1.99 ± 0.13a	2.08 ± 0.09a	1.85 ± 0.10a	2.01 ± 0.31a	0.45	1.85	0.238
Leaf total K (g kg^-1^)	4.90 ± 0.46a	6.04 ± 0.45a	6.29 ± 1.03a	5.82 ± 0.54a	0.48	2.48	0.135
Fruit parameters							
Single fruit weight (g)	257.85 ± 24.10b	254.97 ± 2.26b	294.87 ± 4.12a	298.02 ± 6.96a	0.79	10.29	**0.008**
Fresh weight (g)	115.65 ± 10.13c	129.74 ± 9.85b	150.37 ± 15.66a	140.18 ± 5.65ab	0.8	15.35	**0.003**
Dry weight (g)	21.29 ± 1.96b	21.58 ± 2.37b	25.24 ± 1.91a	25.20 ± 2.76a	0.71	7.89	**0.017**
Dry matter (g)	18.41 ± 0.48a	16.60 ± 0.62b	16.82 ± 0.54ab	17.94 ± 1.23ab	0.59	3.76	0.059
Soluble solids (%)	15.60 ± 0.61a	14.63 ± 0.42b	14.37 ± 0.31b	15.22 ± 0.87ab	0.62	5.22	**0.041**
Malic acid (%)	0.33 ± 0.08a	0.25 ± 0.03ab	0.26 ± 0.01ab	0.25 ± 0.04b	0.51	3.10	0.111
Solid acid ratio	50.33 ± 11.85b	59.34 ± 7.09ab	56.00 ± 2.85ab	62.58 ± 4.60a	0.47	2.50	0.157

^1^ CK, control, no chemical fertilizer and biogas slurry; CF, 100% chemical nitrogen (N) fertilizer; CBS and BS, biogas slurry replacing 50% and 100% of chemical N fertilizer, respectively.

^2^Results are presented as mean ± SD (standard deviation). Different lowercase letters within a row indicate significant differences at the p < 0.05 level among the treatments.

The bold values indicate significant difference at the p < 0.05 level.

### 3.2 Soil bacterial community diversity and composition

**Figure 1:**
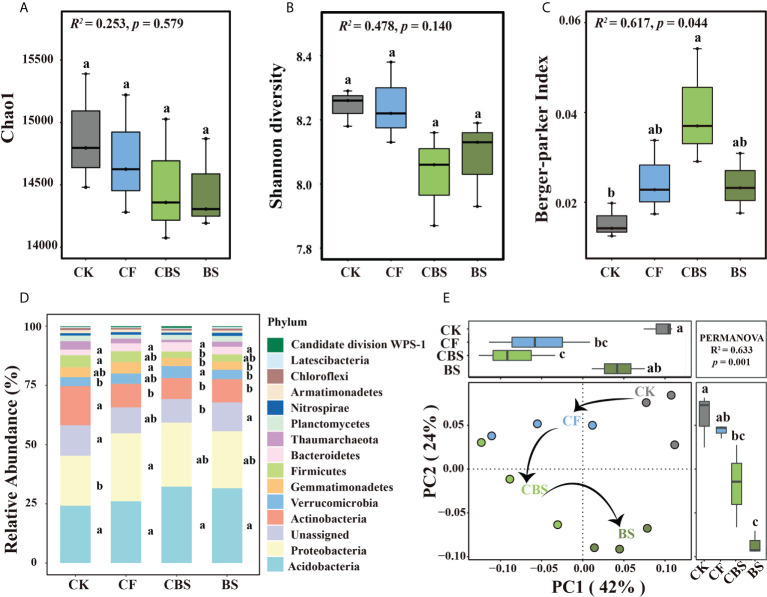
Soil bacterial community diversity **(A–C)** and composition **(D, E)** under different treatments. CK, control, no chemical fertilizer and biogas slurry; CF, 100% chemical nitrogen (N) fertilizer; CBS and BS, biogas slurry replacing 50% and 100% of chemical N fertilizer, respectively. Different lowercase letters indicate significant differences at the p < 0.05 level among the treatments.

Soil bacterial alpha diversity, indicated by chao1 and Shannon diversity, was not significantly different among the four treatments (**Figures 1A, B**). The Berker-parker index in CBS was significantly higher than that in CK treatment (p < 0.05, **Figure 1C**). In all samples, the dominant phyla (relative abundance > 1%) were Acidobacteria, Proteobacteria, Actinobacteria, Verrucomicrobia, Gemmatimonadetes, Firmicutes, Bacteroidetes, Planctomycetes, Nitrospirae, Thaumarchaeota and Armatimonadetes, which accounted for 86.3% of all observed phyla (**Figure 1D**). In addition, 24 other rarer phyla were identified and unassigned 11.4% OTUs. As compared with the CK treatment, the relative abundance of Proteobacteria and Verrucomicrobia increased by 5-36% in the fertilization treatments, whereas the relative abundance of Actinobacteria, Firmicutes, Thaumarchaeota and Armatimonadetes decreased with the rate of using biogas slurry as a substitute for chemical N fertilizer (p < 0.05, **Figure 1D**). The relative abundance of Gemmatimonadetes was significantly higher in CF than in CBS and BS treatments, while the lowest proportion of Nitrospirae was found in CBS treatment. PCoA and PERMANOVA were used to assess the changes in bacterial community composition affected by the different levels of biogas slurry as a substitute for chemical N fertilizer. The first two principal coordinates explained 66% of the total variation in bacterial community composition (**Figure 1E**). The four treatments were distinctly separated from each other in the four quadrants along the increasing rate of chemical N fertilizer replacing by biogas slurry (p = 0.001).

**Figure 2:**
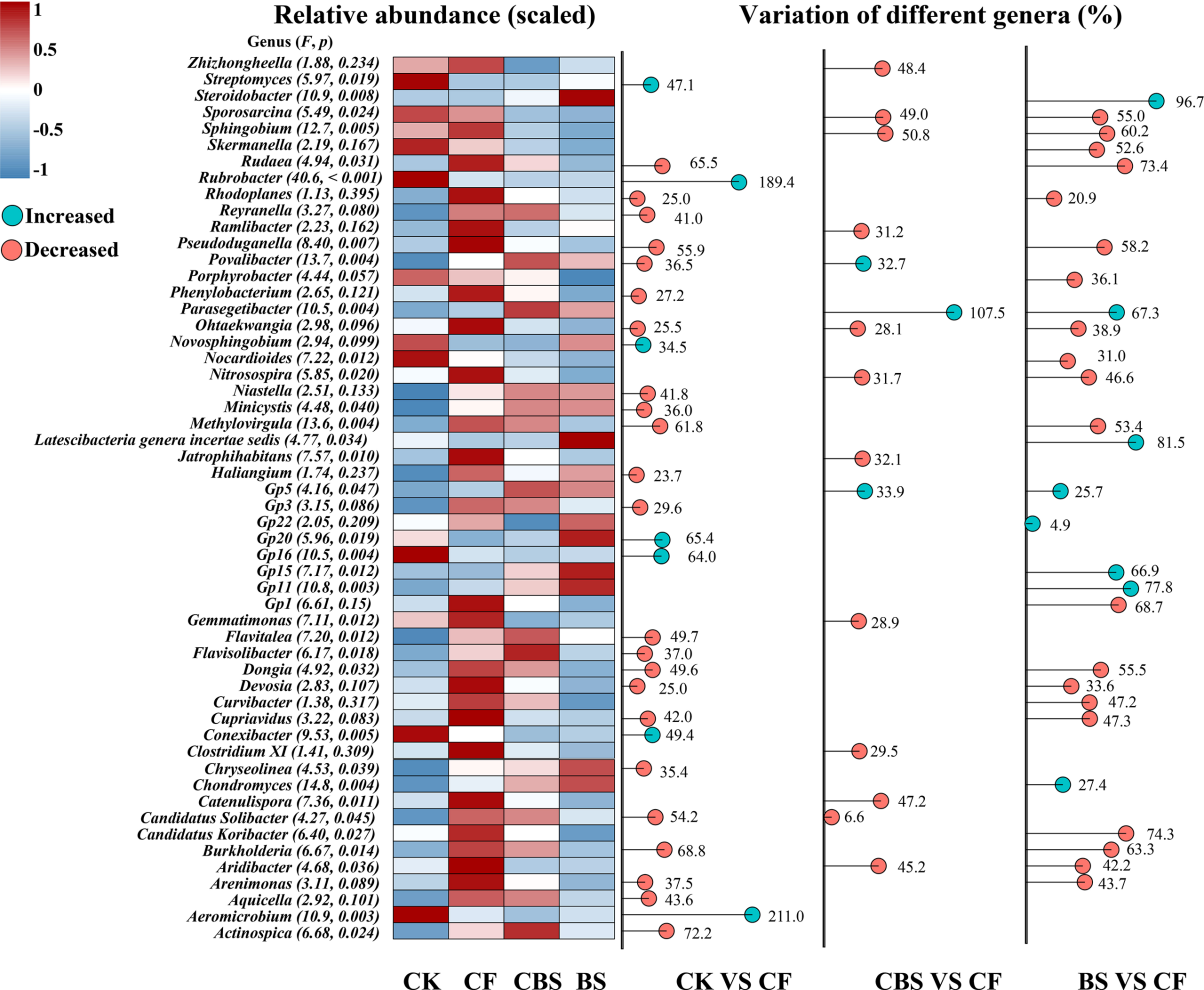
Changes of different bacteria taxa in different treatment groups (CF VS CK, CBS VS CF, and BS VS CF). CK, control, no chemical fertilizer and biogas slurry; CF, 100% chemical nitrogen (N) fertilizer; CBS and BS, biogas slurry replacing 50% and 100% of chemical N fertilizer, respectively.

Soil bacterial communities at different taxonomic ranks (phylum, class, order, family, and genus) showed distinct features through phylogenetic analysis following the increasing rate of chemical N fertilizer replacing by biogas slurry (**Table S1**). Bacterial community was significantly altered by the increasing rate of biogas slurry application (i.e., CF VS CK, 0%; CBS VS CF, 50%; BS VS CF, 100%) (**Figure 2**). For example, compared with the CK treatment, CF addition significantly increased (p < 0.05) the relative abundances of 23 bacterial genera (**Figure 2**). In addition, the relative abundances of genera *Parasegetibacter*, *Gp5*, and *Povalibacter* were significantly higher (p < 0.05) in CBS treatments than in CF treatment, while those of eight genera (including *Steroidobacter*, *Latescibacteria_genera_incertae_sedis*, *Gp11*, *Parasegetibacter*, *Gp15*, *Chondromyces*, *Gp5*, and *Gp22*) were significantly higher (p < 0.05) in BS treatment than in CF treatment (**Figure 2**).

### 3.3 Soil bacterial functional groups

**Figure 3:**
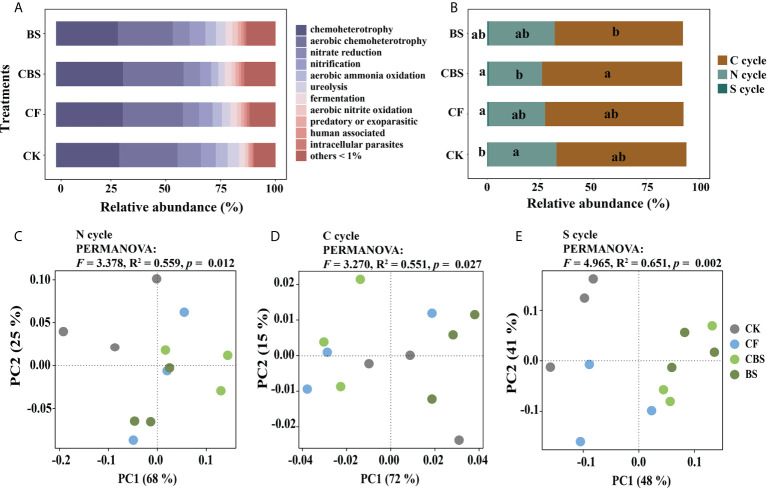
Soil bacterial functional profile **(A)** and functional group composition related to nitrogen (N), carbon (C), and sulfur (S) cycle **(B–E)** in different treatments. CK, control, no chemical fertilizer and biogas slurry; CF, 100% chemical nitrogen (N) fertilizer; CBS and BS, biogas slurry replacing 50% and 100% of chemical N fertilizer, respectively. Different lowercase letters indicate significant differences at the p < 0.05 level among the treatments.

FAPROTAX database was used to annotate bacterial function, and obtained a total of 81 functional groups (**Table S3**) which including 11 most abundant functional groups (relative abundance > 1%, **Figure 3A**). The functional group structure of bacteria was significantly regulated by the biogas slurry addition (**Figure 3B**). In addition, the functional groups related to N, C, and S cycles were significantly affected by the replacement of biogas slurry for chemical N fertilizer, such as chemoheterotrophy, aerobic nitrite oxidation, nitrate reduction, cellulolysis, nitrate respiration, etc. (**Figure 3A**, **Table S2**). We further used PCoA analysis based on Bray-Curtis distance to reflect the structure of N, C, and S cycles related functional groups, respectively, and the differences among the four treatments were tested using PERMANOVA analysis (**Figures 3C–E**). The results showed that the fertilizer treatments harbored different N and S cycles related functional profiles than the CK treatment, while the C cycle related functional groups in the CF and CBS treatments were separated with CK and BS.

STAMP analysis was used to determine the significant differences in functional groups following the increment of replacing rate by biogas slurry for chemical N fertilizer (CK VS CF, CBS VS CF, and BS VS CF, **Figure S1**). The relative abundances of several functional groups in CF treatment were significantly higher than CK (p < 0.05) which includes aerobic nitrite oxidation, anoxygenic photoautotrophy, anoxygenic photoautotrophy S oxidizing, fumarate respiration, photoheterotrophy, intracellular parasites, predatory or exoparasitic photoautotrophy and xylanolysis. When the 50% of chemical N fertilizer was replaced by biogas slurry, the relative abundance of reductive acetogenesis was increased while that of manganese respiration was reduced in CBS than in CF. In addition, the BS treatment significantly increased the relative abundance of aerobic nitrite oxidation, denitrification, nitrate denitrification, nitrite respiration and nitrous oxide denitrification than those in CF treatment (p < 0.05). Based on the FAPROTAX results, the associated bacteria of the different functional groups could be correspondingly assigned to *Nitrospira* (aerobic nitrite oxidation), *Arthrobacter*, *Bacillus* (nitrate reduction), and *Chryseolinea*, *Opitutus* (xylanolysis) (**Table S3**). These results suggest that the variation of bacterial community associated with functional changes in response to the replacement of chemical N fertilizer by biogas slurry.

### 3.4 Correlations among soil and plant parameters with soil bacterial community and function

**Figure 4:**
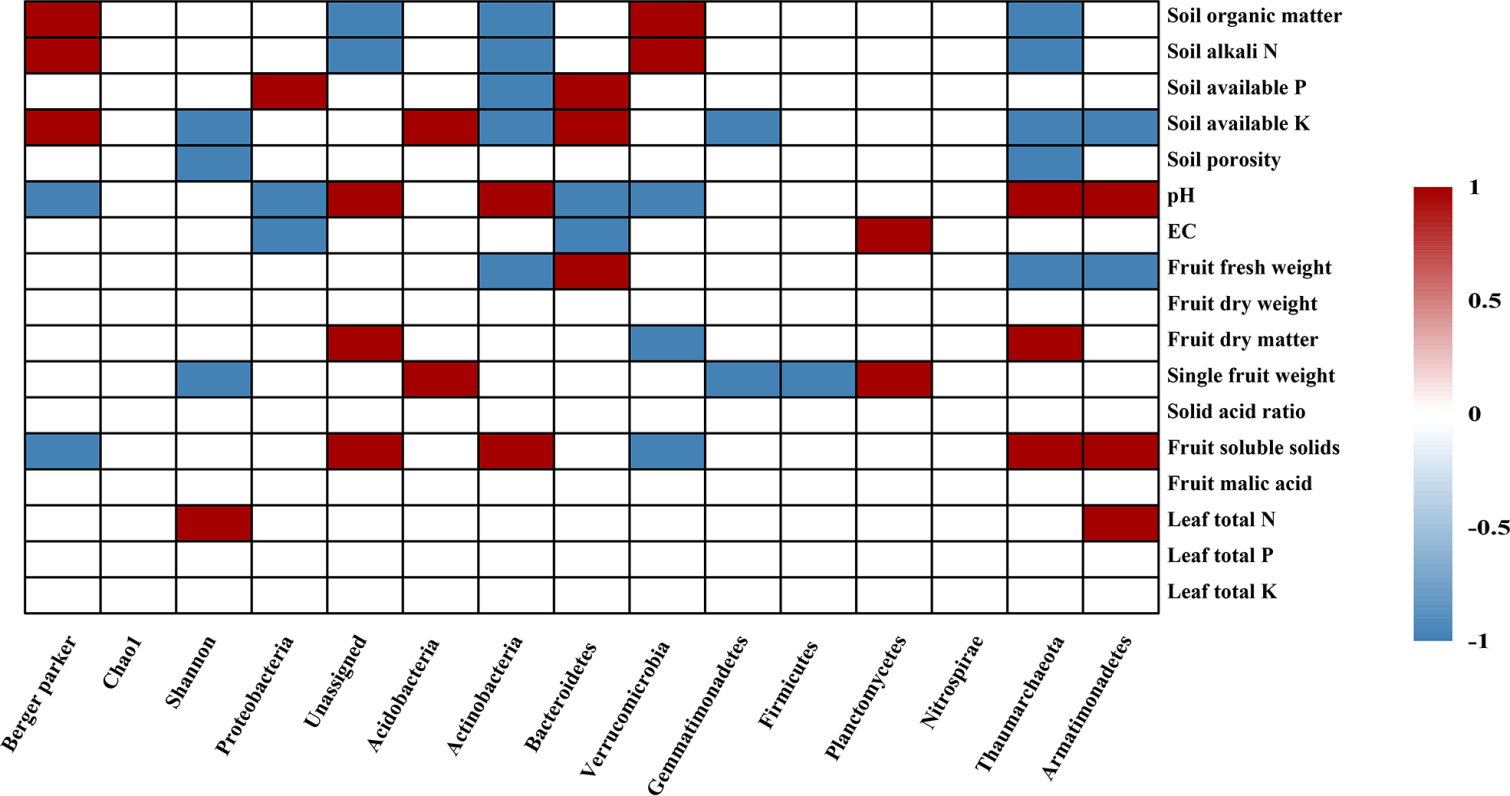
Significant correlations of soil bacterial diversity and composition with soil physico-chemical parameters, apple leaf nutrients and fruit parameters under different treatments. Red and blue squares in the heatmap represent the significant Spearman correlation coefficient at p < 0.05 level, and white square represent insignificant, respectively.

**Figure 5:**
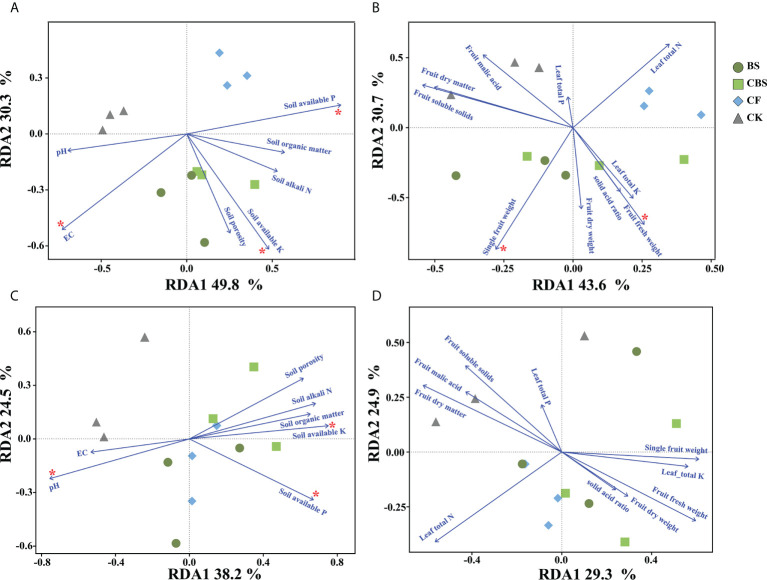
Redundancy analysis showing the relationships between soil and plant parameters with the different genera community composition **(A, B)** and functional structure **(C, D)** of bacteria in different treatments. CK, control, no chemical fertilizer and biogas slurry; CF, 100% chemical nitrogen (N) fertilizer; CBS and BS, biogas slurry replacing 50% and 100% of chemical N fertilizer, respectively.

When considered across all soil samples, Spearman correlation coefficients were determined to evaluate the correlations among soil bacterial community and functional structure with soil and plant parameters. The Berker-parker index value had significant correlation with the contents of soil organic matter, soil alkali N, soil porosity and leaf total N and K, fruit fresh weight, pH, fruit dry matter and soluble solids (p < 0.05, **Figure 4**). The Chao1 value was positively correlated with leaf total N content and fruit malic acid, while the Shannon diversity index had a positive relation with leaf total N content but negative correlation with soil available K and single fruit weight (p < 0.05, **Figure 4**). The relationship of various soil and plant properties with bacterial community composition and functional structure were further investigated using RDA analysis (**Figure 5**). These investigations revealed that soil and plant parameters together explain 80.1% and 74.3%, 62.7% and 54.2% of the observed variations in bacterial community composition (**Figures 5A, B**) and functional structure (**Figures 5C, D**), respectively. And the results showed that soil available P and K, EC, single fruit weight, and fruit fresh weight were significantly related to the changes in bacterial community composition (p < 0.05), while soil organic matter, soil available P and pH were the most important soil factors affecting the bacterial functional structure (p < 0.05) (**Figure S2**).

**Figure 6:**
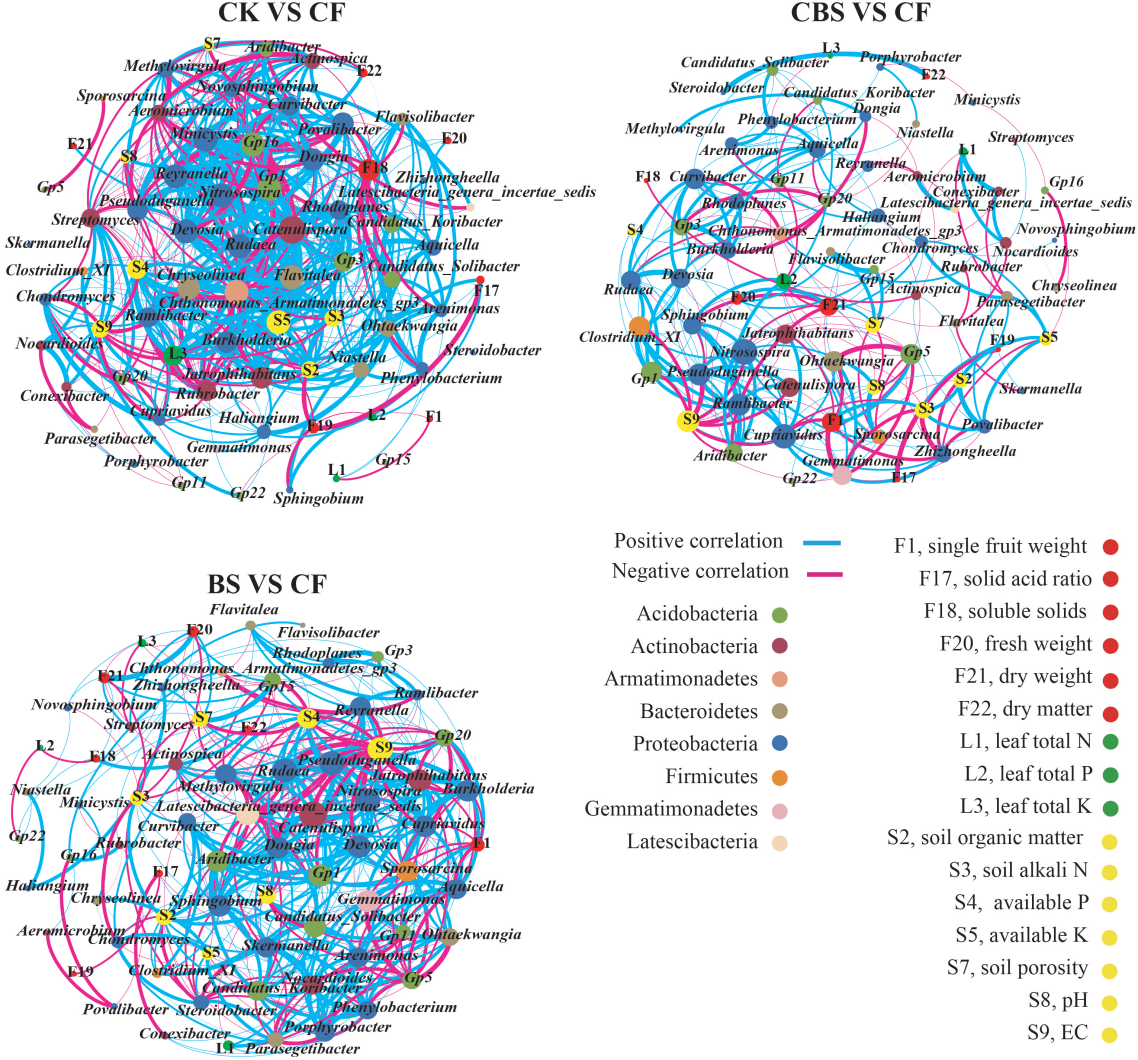
Changes of relations among soil bacterial genera with soil physico-chemical parameters, leaf nutrients and fruit parameters of apple following the increment of replacing rate by biogas slurry for chemical N fertilizer. In the networks, the nodes represent the bacterial genera or soil and plant parameters. The blue links indicate the significant positive correlations between the two individual nodes, whereas the red links indicate negative correlations. The thickness of the edge represents the magnitude of correlations. CK, control, no chemical fertilizer and biogas slurry; CF, 100% chemical nitrogen (N) fertilizer; CBS and BS, biogas slurry replacing 50% and 100% of chemical N fertilizer, respectively.

To reveal the changing relationship patterns of different genera with soil and plant variables, Spearman’s correlation coefficients were conducted and visualized by using network in different comparison groups, respectively (**Figure 6**). In the networks, the order of total degrees in the three comparison group networks was CF VS CK (590) > BS VS CF (493) > CBS VS CF (258), however, the core genera which was having significant correlations with soil and plant parameters were varied. For example, some genera were consistently correlated with most of plant and soil parameters in the three groups (*Carenulispora*, *Chondromyces*, *Cupriavidus*, *Nitrosospira*, *Pseudoduganella*, *Rudaea*, et al.), while the genera *Candidatus_Solibacter*, *Dongia*, *Methylovirgula* and *Streptomyces* were found in the groups of CF VS CK and CBS VS CF, and *Gemmatimonas*, *Gp15*, and *Parasegetibacter* in the groups of CBS VS CF and BS VS CF, respectively (**Figure 6**). In addition, we also determined the relationships between the relative abundances of significantly different functional groups with plant and soil parameters following the increment of biogas slurry application (**Table S4**). The functional groups were mostly correlated with soil parameters. For example, functional groups of xylanolysis, photoheterotrophy, anoxygenic photoautotrophy, photoautotrophy, fumarate respiration were negatively correlated with pH in CF VS CK comparing group. And in BS VS CF comparing group, functional groups of aerobic nitrite oxidation, nitrite respiration, denitrification were positively correlated with pH and EC. In contrast, pH and EC represented a negative correlation with aerobic anoxygenic phototrophy.

## 4 Discussion

### 4.1 Leaf nutrients, apple fruit and soil properties affected by the substitution of biogas slurry for chemical N fertilizer

In the present study, compared with CK and CF treatments, the application of biogas slurry solely or combined with chemical N fertilizer improved soil and plant status, such as the contents of soil organic matter and available P and K, soil porosity, and fruit weight (single fruit, fresh or dry fruit pulp) (**Table 2**). Many studies have concluded that the application of raw biogas slurry or combined with other fertilizer could improve soil properties and plant growth, which contributed to the functional microorganism, active ingredients and available nutrients in biogas slurry (Wentzel et al., 2015; Wang et al., 2019; Ren et al., 2020). In addition, we observed that the contents of soil organic matter, alkali N and available K, soil porosity, and fruit weight first increased and then decreased following the increasing levels of biogas slurry application, indicating that the benefits of biogas slurry to soil and plant were dose-dependent, which were similar with previous studies (Wang et al., 2019; Xu et al., 2019; Zhang et al., 2021). As there was the same pure nitrogen addition in this study CBS treatments provided multi-types of nutrients for crop growth, and the proper application ratio were reported that could reduce the loss of carbon and nitrogen by leaching, runoff and gas emission (Michos et al., 2012; Young et al., 2021). In addition, the application of CF and CBS significantly reduced soil pH. This result contrasts with previous studies, which have reported that organic amendments, such as bio-organic fertilizer and biogas slurry, could increase soil pH by improving soil acid buffering capacity (Xu et al., 2019; Zhang et al., 2021). However, other studies also indicated that the positive effects of organic material on pH vanished when the soil initial pH was higher than the organic materials application (Zhang et al., 2016). The value of soil pH in CK treatment was 7.84 which was higher than that in the raw biogas slurry (7.65), as a result there observed a decline of soil pH in CBS and BS treatments. Similar results were also observed in early studies (Zhang et al., 2016; Yang et al., 2020; Zhang et al., 2021). Thus, the substitution of biogas slurry for chemical fertilizer at a proper ratio (50% in the present study) effectively improved apple plant growth and soil nutrients.

### 4.2 Changes of soil bacterial community affected by the substitution of biogas slurry for chemical N fertilizer

Soil microbial diversity is one of the effective indicators of soil quality (Bünemann et al., 2018). Our results showed that soil bacterial Berger-parker dominance index was significantly increased by the CBS treatment in comparison to that in CK treatment. This result suggested that application of biogas slurry combined with chemical N fertilizer enriched specific bacterial taxa. Generally, organic amendments could stimulate soil microbial biomass and activity attributing to increased organic matter and available nutrients, however, some substances in organic amendments may produce suppressive effects on some specialized microbial groups (Insam et al., 2015; Cao et al., 2016; Tang et al., 2021). According to the Spearman’s correlation results, the contents of soil organic matter, alkali N and available P positively affected bacterial Berger-parker dominance index, while soil pH showed negative effects on that. Studies have shown that biogas slurry application had positive effects on soil bacterial alpha diversity (eg., Chao1, Shannon diversity), but in the present study there was no significant variance observed which may due to the differences in application ratio and management time (Xu et al., 2019; Liu et al., 2021; Tang et al., 2021).

Soil bacterial community play critical roles in regulating soil nutrients and plant growth, and in turn they response rapidly and have strong links with environmental variation resulting from biogas slurry addition (Xu et al., 2019; Ren et al., 2020; Tang et al., 2021; Zhang et al., 2021). The results of PCoA analysis and community dissimilarity test revealed that soil bacterial community varied among the four fertilizer regimes and the relative abundance of dominant phyla (> 1%) differed notably among the four treatments. For example, with the ratio of biogas slurry and chemical N fertilizer increased, the relative abundance of Proteobacteria and Verrucomicrobia increased and then decreased. This was supported by the previous observations that Proteobacteria was copiotrophic and could colonize well in nutrient-rich environment (Xu et al., 2019; Zhang et al., 2021; Dang et al., 2022) and Verrucomicrobia was positively correlated with soil organic matter content and critical in increasing plant photosynthesis efficiency and material accumulation (Xu et al., 2019; Duan et al., 2022), which was consistent with the improved soil nutrients and plant parameters in CBS treatment (**Table 2**). The phyla Actinobacteria, Gemmatimonadetes, and Firmicutes have been reported taking role in the process of carbon turnover especially for degrading recalcitrant compounds (Ali et al., 2019; Xu et al., 2019). The relative abundances of Actinobacteria, Gemmatimonadetes, and Firmicutes were decreased after chemical N fertilizer replaced by biogas slurry especially in CBS. These results suggested that the CBS treatment improved soil nutrient availability and increased soil carbon storage through regulating specific bacterial groups. According to the RDA analysis, soil available P and K and EC were the main factors affecting soil bacterial community composition which was consistent with the findings of previous studies (Chen et al., 2020; Li et al., 2021; Liu et al., 2021). And apple single fruit weight and fruit fresh weight also influenced the soil bacterial community in the present study. These results demonstrated that the substitution of biogas slurry with chemical fertilizer regulated soil bacterial community by the improvement of soil nutrient availability and apple plant growth.

Furthermore, different rate substitution of organic amendment for chemical N fertilizer altered the microbial community composition by regulating specific taxa relative abundance (Xu et al., 2019; Liu et al., 2021). Our results demonstrated that the treatment with 50% biogas slurry replacing for chemical N fertilizer significantly increased the relative abundances of genera *Parasegetibacter*, *Gp5*, and *Povalibacter*, while genera *Steroidobacter*, *Latescibacteria_genera_incertae_sedis*, *Gp11*, *Gp15*, *Chondromyces*, and Gp22 were positively correlated the biogas slurry addition in BS treatment. Previous studies had shown that genera *Parasegetibacter* and *Steroidobacter* were beneficial microbes and could cause a decrease in extracellular antibiotic resistance genes in the soil, and could be activated by organic application (Semenov et al., 2020; Milkereit et al., 2021; Zhang et al., 2021; Zhu et al., 2021). It was also reported that Acidobacteria could promote crop growth, and higher abundances of Acidobacteria *Gp5* and *Gp7* often existed in disease suppressive soils (Shen et al., 2015; Xu et al., 2019). Together with the correlations among differential genera with plant and soil parameters in the three comparison groups, these findings indicated that biogas slurry addition could enhance plant growth by improving soil nutrients and enriching beneficial microbes.

### 4.3 Changes of soil bacterial functional structure affected by the substitution of biogas slurry for chemical N fertilizer

Previous studies have shown that the substitution of chemical fertilizer with organic fertilizer led to the changes in microbial community composition and functional groups (Li et al., 2021; Liu et al., 2021; Chen et al., 2022; Dang et al., 2022). Some bacterial functional groups (e.g. Chemoheterotrophy, Nitrate reduction, Aerobic nitrite oxidation) relating to C and N cycles were found changing significantly with the substitution of chemical N fertilizer with biogas slurry (**Figure 3**, **Table S2**), indicating that the changing of bacterial community structure might affect soil nutrients and element cycling (Insam et al., 2015; Ren et al., 2020; Cai et al., 2021; Tang et al., 2021). Specifically, aerobic nitrite oxidation, nitrogen respiration, nitrate respiration and nitrite respiration functional groups may play key roles in nutrient cycling following the increment of biogas slurry substitution proportion, while chemoheterotrophy, cellulolysis, and nitrogen fixation functional groups may be more important in CBS treatment. Due to the substitution of chemical N fertilizer with biogas slurry, the characteristics of soil and apple plant were altered and differed from CK and CF, and according to the RDA results showed that soil nutrients regulated the bacterial functional structure. Therefore, due to the differences in available resources, the functional groups of bacterial community showed different patterns following the increasing ratio of biogas slurry application in the three comparison groups (CK VS CF, CBS VS CF, and BS VS CF).

## 5 Conclusions

Through a comparison of soil bacterial community composition, functional structure, and their relationships with apple plant growth and soil parameters under different treatments, we found that biogas slurry was more suitable than chemical fertilizer for increasing soil nutrition and improving apple plant growth and fruit parameters, especially in CBS treatment (with 50% chemical N fertilizer substituted by biogas slurry). Biogas slurry application influenced soil bacterial community dominance and composition, and increased the relative abundance of some beneficial taxa and functional groups related to C and N cycles. The variations in soil available P and K, pH and EC were identified as soil factors, which were having a high potential for regulating soil bacterial specific taxa and functional groups. Furthermore, some bacterial taxa playing specific function in soil could improve apple plant growth by modulating soil properties. Above all, with consideration of apple plant growth and sustainability of soil ecosystem, a proper ratio of biogas slurry and chemical fertilizer (50% in the present study) is recommended for apple orchard.

## Data availability statement

The datasets presented in this study can be found in online repositories. The names of the repository/repositories and accession number(s) can be found below: NCBI - PRJNA 875317.

## Author contributions

CZ and XZ conceived the experiments and designed the study. HZ, YM, JZS, RD, ZY, and JSS performed the experiments and analyzed the data. HZ, YM, FZ, XZ, and CZ drafted the manuscript, which was reviewed by all authors. All authors contributed to the article and approved the submitted version.

## Funding

This research was funded by the National Key Research and Development Program of China (2016YFD0201100), the Science and Technology Research Project of Colleges and Universities in Hebei Province of China (ZD2019135), the Key Research and Development Program of Hebei Province of China (22326802D), the Earmarked Fund for Hebei Apple Innovation Team of Modern Agroindustry Technology Research System (HBCT2021100409;HBCT2021100210)and Science and Technology Service Network Initiative (STS) of Chinese Academy of Sciences (KFJ-STS-QYZD-160).

## Conflict of interest

The authors declare that the research was conducted in the absence of any commercial or financial relationships that could be construed as a potential conflict of interest.

## Publisher’s note

All claims expressed in this article are solely those of the authors and do not necessarily represent those of their affiliated organizations, or those of the publisher, the editors and the reviewers. Any product that may be evaluated in this article, or claim that may be made by its manufacturer, is not guaranteed or endorsed by the publisher.
