# Supplementary material for: Changes in soil bacterial community and functions by substituting chemical fertilizer with biogas slurry in an apple orchard
Source: Front Plant Sci. 2022 Sep 20;13:1013184. doi: 10.3389/fpls.2022.1013184 (PMC9530944; doi:10.3389/fpls.2022.1013184)
Supplement: Supplementary file 1 [file DataSheet_1.docx]

Supplementary Material

Supplementary Table and Figure captions, Table S3, S4, Figure S1 and S2 are uploaded separately on submission.

Table S1. Significantly increased taxa by relative abundance group in 100% chemical N fertilizer, biogas slurry replacing chemical N fertilizer at 50% (CBS) and 100% (BS) soils.

Table S2. Effects of the replacement of biogas slurry for chemical N fertilizer on soil bacterial functional groups related to N, C and S cycle.

Table S3. Summary of functional group assignments by FAPROTAX method.

Table S4. Changes of relations among soil bacterial functional groups with soil physico-chemical parameters, leaf nutrients and fruit parameters of apple following the increment of replacing rate by biogas slurry for chemical N fertilizer. Table S4A, correlation coefficient, r; Table S4B, significant value, p. CK, control, no chemical fertilizer and biogas slurry; CF, 100% chemical nitrogen (N) fertilizer; CBS and BS, biogas slurry replacing 50% and 100% of chemical N fertilizer, respectively.

Figure S1. Changes of functional groups with significantly different relative abundance fol-lowing the increment of replacing rate by biogas slurry for chemical N fertilizer.

Figure S2. Contribution (r^2^) of plant (A and B) and soil (C and D) variables to bacterial community composition and functional structure.

Table S1. Significantly increased taxa by relative abundance group in 100% chemical N fertilizer, biogas slurry replacing chemical N fertilizer at 50% (CBS) and 100% (BS) soils.

| **Taxa** | Abundance group, % | Number of taxa (relative abundance, %) | | | | CK to CF | | | CF to CBS | | | CF to BS | | |
| --- | --- | --- | --- | --- | --- | --- | --- | --- | --- | --- | --- | --- | --- | --- |
|  |  | CK | CF | CBS | BS | Increase | CK | CF | Increase | CF | CBS | Increase | CF | BS |
| **Phylum** | >1 | 12(97.9) | 12(97.8) | 10(95.7) | 11(96.8) | 2(8.40) | 23.4b | 31.8a | 0(0.00) | 0.00c | 0.00c | 0(0.00) | 0.00c | 0.00c |
|  | 0.1-1 | 4(1.8) | 8(2.1) | 8(4.1) | 5(2.9) | 2(0.15) | 0.17b | 0.32a | 0(0.00) | 0.00c | 0.00c | 0(0.00) | 0.00c | 0.00c |
|  | 0.01-0.1 | 7(0.3) | 3(0.1) | 5(0.2) | 7(0.3) | 0(0.00) | 0.00a | 0.00a | 0(0.00) | 0.00a | 0.00a | 0(0.00) | 0.00a | 0.00a |
|  | <0.01 | 11(0.0) | 11(0.0) | 11(0.0) | 11(0.0) | 1(0.003) | 0.001b | 0.004a | 0(0.00) | 0.00b | 0.00b | 0(0.00) | 0.00b | 0.00b |
|  | **Sum** | **34(100)** | **34(100)** | **34(100)** | **34(100)** | **5(8.55)** | **23.6b** | **32.1a** | **0(0.00)** | **0.00c** | **0.00c** | **0(0.00)** | **0.00c** | **0.00c** |
| **Class** | >1 | 16(92.6) | 16(91.9) | 15(91.3) | 16(92.5) | 3(6.82) | 17.5b | 24.3a | 0(0.00) | 0.00c | 0.00c | 0(0.00) | 0.00c | 0.00c |
|  | 0.1-1 | 16(6.6) | 17(7.4) | 18(8.1) | 17(6.8) | 3(0.94) | 1.19b | 2.13a | 1(0.17) | 0.56c | 0.73c | 1(0.05) | 0.08d | 0.13d |
|  | 0.01-0.1 | 16(0.7) | 16(0.6) | 14(0.6) | 14(0.6) | 0(0.00) | 0.00c | 0.00c | 0(0.00) | 0.00c | 0.00c | 2(0.04) | 0.09b | 0.13a |
|  | <0.01 | 21(0.1) | 21(0.1) | 21(0.1) | 23(0.1) | 2(0.006) | 0.001b | 0.01a | 0(0.00) | 0.00c | 0.00c | 0(0.00) | 0.00c | 0.00c |
|  | **Sum** | **69(100)** | **70(100)** | **68(100)** | **70(100)** | **8(7.76)** | **18.7b** | **26.4a** | **1(0.17)** | **0.56a** | **0.73a** | **3(0.09)** | **0.17a** | **0.26a** |
| **Order** | >1 | 17(92.1) | 17(91.1) | 15(90.0) | 17(92.4) | 3(3.38) | 4.69b | 8.07a | 0(0.00) | 0.00c | 0.00c | 0(0.00) | 0.00c | 0.00c |
|  | 0.1-1 | 21(7.3) | 20(8.1) | 24(9.4) | 20(6.8) | 5(1.10) | 1.41b | 2.51a | 0(0.00) | 0.00c | 0.00c | 0(0.00) | 0.00c | 0.00c |
|  | 0.01-0.1 | 18(0.6) | 19(0.7) | 16(0.5) | 19(0.7) | 1(0.02) | 0.05c | 0.06b | 1(0.02) | 0.06b | 0.08a | 0(0.00) | 0.00d | 0.00d |
|  | <0.01 | 29(0.1) | 31(0.1) | 29(0.1) | 31(0.1) | 1(0.003) | 0.001b | 0.004a | 0(0.00) | 0.00b | 0.00b | 0(0.00) | 0.00b | 0.00b |
|  | **Sum** | **85(100)** | **87(100)** | **84(100)** | **87(100)** | **11(4.50)** | **6.2b** | **10.6a** | **1(0.02)** | **0.06c** | **0.08c** | **0(0.00)** | **0.00c** | **0.00c** |
| **Family** | >1 | 12(83.9) | 12(82.8) | 9(82.0) | 11(84.4) | 2(2.15) | 2.4b | 4.5a | 0(0.00) | 0.00c | 0.00c | 0(0.00) | 0.00c | 0.00c |
|  | 0.1-1 | 48(13.5) | 49(14.7) | 51(15.5) | 43(12.7) | 11(1.82) | 3.2b | 5.0a | 0(0.00) | 0.00c | 0.00c | 0(0.00) | 0.00c | 0.00c |
|  | 0.01-0.1 | 62(2.3) | 59(2.2) | 57(2.2) | 67(2.7) | 2(0.03) | 0.05c | 0.08c | 1(0.02) | 0.04c | 0.06c | 2(0.14) | 0.62b | 0.76a |
|  | <0.01 | 86(0.3) | 91(0.3) | 93(0.3) | 90(0.3) | 0(0.00) | 0.00a | 0.00a | 0(0.00) | 0.00a | 0.00a | 0(0.00) | 0.00a | 0.00a |
|  | **Sum** | **208(100)** | **211(100)** | **210(100)** | **211(100)** | **15(4.00)** | **5.6b** | **9.6a** | **1(0.02)** | **0.04d** | **0.06d** | **2(0.06)** | **0.62c** | **0.76c** |
| **Genus** | >1 | 13(79.9) | 11(76.4) | 8(75.1) | 10(78.1) | 0(0.00) | 0.00a | 0.00a | 0(0.00) | 0.00a | 0.00a | 0(0.00) | 0.00a | 0.00a |
|  | 0.1-1 | 52(13.2) | 64(17.4) | 61(18.5) | 53(15.3) | 16(1.77) | 2.7b | 4.5a | 1(0.17) | 0.54e | 0.71de | 5(0.49) | 0.91c | 1.40c |
|  | 0.01-0.1 | 171(5.7) | 169(5.1) | 150(5.2) | 157(5.4) | 15(0.31) | 0.32b | 0.63a | 2(0.07) | 0.09e | 0.16d | 4(0.10) | 0.13de | 0.23c |
|  | <0.01 | 406(1.2) | 396(1.1) | 432(1.3) | 432(1.2) | 5(0.011) | 0.004c | 0.015b | 0(0.00) | 0.00c | 0.00c | 6(0.02) | 0.01b | 0.03a |
|  | **Sum** | **642(100)** | **640(100)** | **651(100)** | **652(100)** | **36(2.09)** | **3.08b** | **5.17a** | **3(0.15)** | **0.63e** | **0.86de** | **15(0.62)** | **1.05d** | **1.67c** |

Different letters following relative abundance indicate significant difference in the same row (*p* < 0.05); The “Increase” column were the relative abundance difference between CF VS CK, CBS VS CF, and BS VS CF, respectively; CK, control, no chemical fertilizer and biogas slurry; CF, 100% chemical nitrogen (N) fertilizer; CBS and BS, biogas slurry replacing 50% and 100% of chemical N fertilizer, respectively.

Table S2. Effects of the replacement of biogas slurry for chemical N fertilizer on soil bacterial functional groups related to N, C and S cycle.

| Functional group | Linear mixed effect model | | |
| --- | --- | --- | --- |
|  | F | P | R2 |
| Aerobic ammonia oxidation | 2.30 | 0.177 | 0.464 |
| **Aerobic nitrite oxidation** | **42.7** | **0.000** | **0.932** |
| Anammox | 0.38 | 0.770 | 0.460 |
| Denitrification | 4.00 | 0.052 | 0.600 |
| Nitrate ammonification | 0.41 | 0.748 | 0.134 |
| Nitrate denitrification | 2.91 | 0.101 | 0.522 |
| **Nitrate reduction** | **6.54** | **0.015** | **0.710** |
| **Nitrate respiration** | **4.89** | **0.047** | **0.644** |
| Nitrification | 2.41 | 0.166 | 0.480 |
| Nitrite ammonification | 0.37 | 0.780 | 0.120 |
| Nitrite denitrification | 4.00 | 0.052 | 0.600 |
| **Nitrite respiration** | **4.52** | **0.039** | **0.629** |
| **Nitrogen fixation** | **16.2** | **0.003** | **0.851** |
| **Nitrogen respiration** | **6.20** | **0.029** | **0.695** |
| Nitrous oxide denitrification | 4.00 | 0.052 | 0.600 |
| Ureolysis | 2.60 | 0.147 | 0.494 |
| Aerobic anoxygenic phototrophy | 3.28 | 0.080 | 0.551 |
| Aerobic chemoheterotrophy | 3.33 | 0.077 | 0.556 |
| Aliphatic non methane hydrocarbon degradation | 0.48 | 0.708 | 0.151 |
| Anoxygenic photoautotrophy | 2.55 | 0.129 | 0.489 |
| Anoxygenic photoautotrophy H_2_ oxidizing | 1.09 | 0.409 | 0.290 |
| Anoxygenic photoautotrophy S oxidizing | 2.26 | 0.159 | 0.458 |
| Aromatic compound degradation | 0.47 | 0.713 | 0.182 |
| Aromatic hydrocarbon degradation | 0.17 | 0.914 | 0.072 |
| **Cellulolysis** | **4.54** | **0.039** | **0.630** |
| **Chemoheterotrophy** | **5.31** | **0.026** | **0.666** |
| Fermentation | 0.41 | 0.753 | 0.132 |
| **Fumarate respiration** | **6.45** | **0.016** | **0.707** |
| Hydrocarbon degradation | 0.72 | 0.574 | 0.251 |
| **Hydrogenotrophic methanogenesis** | **4.51** | **0.039** | **0.629** |
| **Ligninolysis** | **4.44** | **0.041** | **0.625** |
| **Methanogenesis** | **4.46** | **0.040** | **0.626** |
| Methanogenesis by CO_2_ reduction with H_2_ | 0.99 | 0.458 | 0.352 |
| **Methanogenesis by reduction of methyl compounds with H_2_** | **4.50** | **0.040** | **0.628** |
| Methanol oxidation | 0.94 | 0.466 | 0.260 |
| Methanotrophy | 0.19 | 0.899 | 0.071 |
| Methylotrophy | 0.94 | 0.479 | 0.264 |
| Photoautotrophy | 2.55 | 0.129 | 0.489 |
| Photoheterotrophy | 2.95 | 0.098 | 0.525 |
| Phototrophy | 2.42 | 0.141 | 0.476 |
| Reductive acetogenesis | 0.70 | 0.576 | 0.209 |
| Xylanolysis | 3.98 | 0.053 | 0.599 |
| Chitinolysis | 3.57 | 0.066 | 0.573 |
| Dark oxidation of sulfur compounds | 2.37 | 0.146 | 0.471 |
| Dark sulfide oxidation | 1.83 | 0.243 | 0.423 |
| Dark sulfur oxidation | 2.76 | 0.134 | 0.510 |
| Dark thiosulfate oxidation | 2.62 | 0.123 | 0.496 |
| **Respiration of sulfur compounds** | **13.7** | **0.002** | **0.837** |
| **Sulfate respiration** | **12.6** | **0.002** | **0.825** |
| Sulfite respiration | 3.04 | 0.093 | 0.533 |
| Sulfur respiration | 0.58 | 0.648 | 0.244 |
| Thiosulfate respiration | 0.90 | 0.481 | 0.253 |
