# Supplementary figures and images for: Changes in soil bacterial community and functions by substituting chemical fertilizer with biogas slurry in an apple orchard
Source: Front Plant Sci. 2022 Sep 20;13:1013184. doi: 10.3389/fpls.2022.1013184 (PMC9530944; doi:10.3389/fpls.2022.1013184)

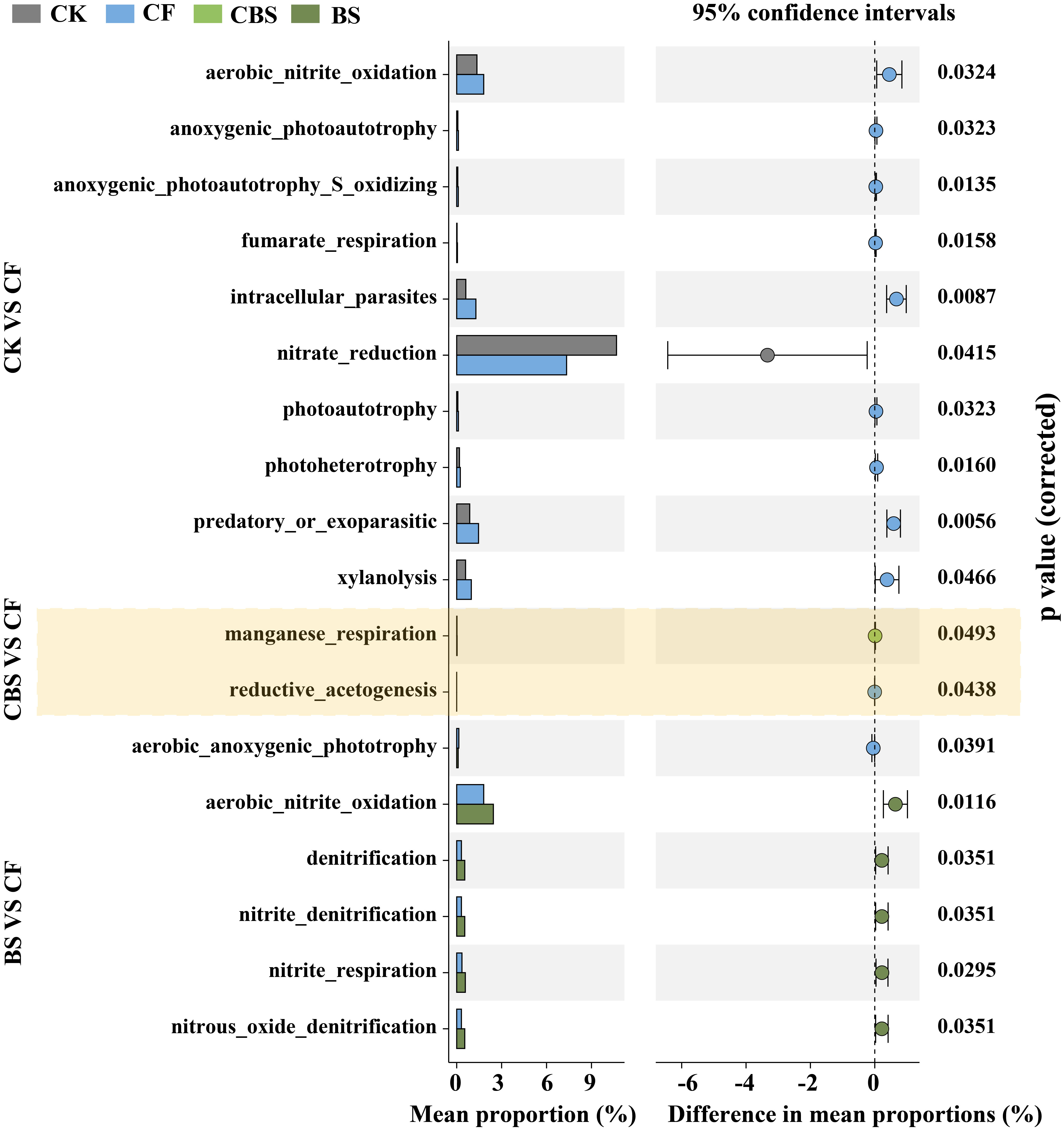

Supplement: Supplementary file 3 [file Image_1.jpeg]

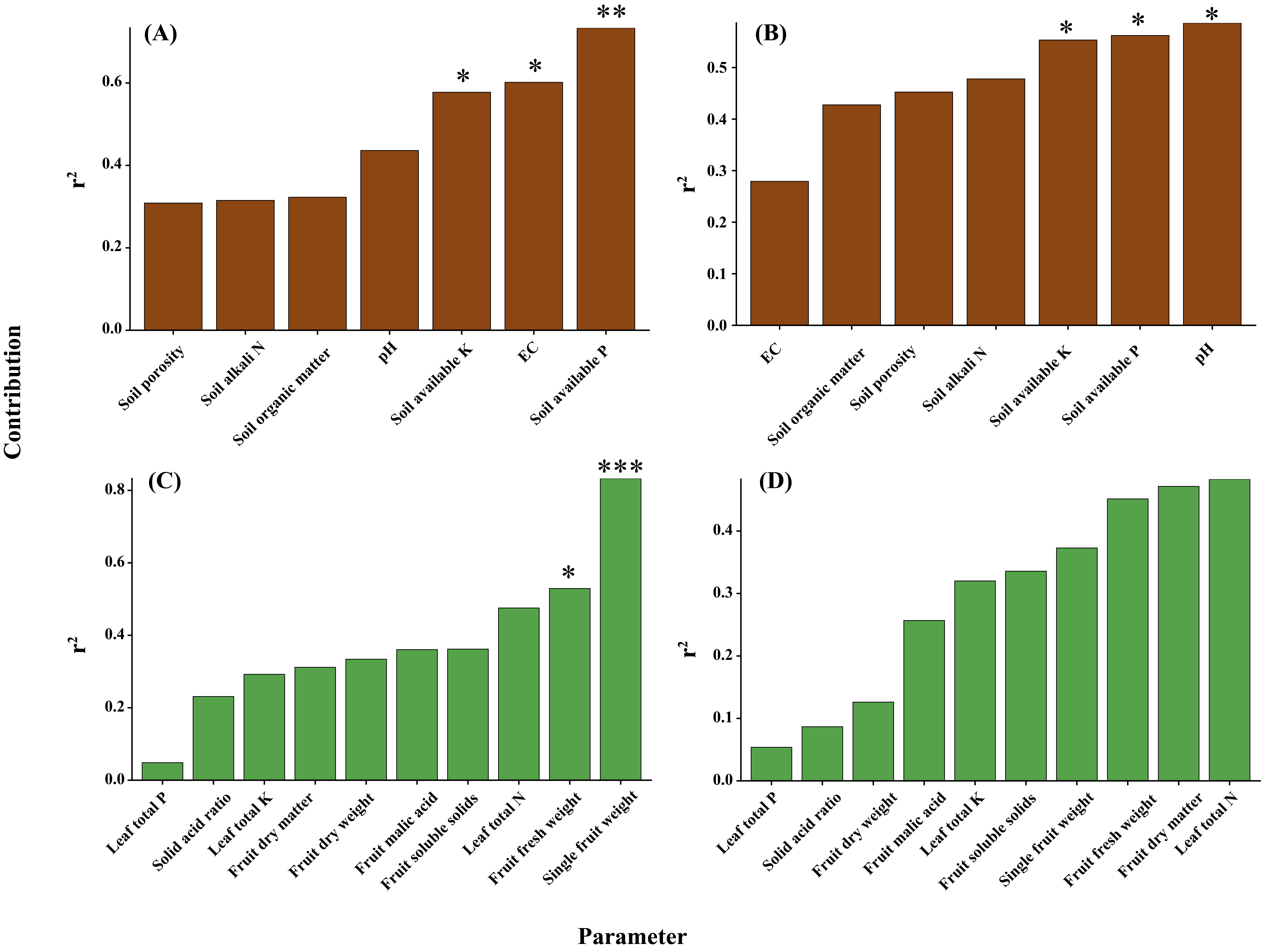

Supplement: Supplementary file 4 [file Image_2.jpeg]
